# The diagnosis values of serum STAT4 and sEng in preeclampsia

**DOI:** 10.1002/jcla.23073

**Published:** 2019-10-19

**Authors:** Luyan Zhang, Xuechun Li, Chengcheng Zhou, Zhengming You, Jianwei Zhang, Guomei Cao

**Affiliations:** ^1^ Department of Laboratory Medicine Ningbo Mingzhou Hospital Ningbo China

**Keywords:** diagnosis, preeclampsia, soluble endoglin, STAT4

## Abstract

**Objective:**

To detect the levels of signal transducer and activator of transcription 4 (STAT4) and soluble endoglin (sEng) in preeclampsia patients and analyze the diagnostic values of STAT4 and sEng in preeclampsia.

**Methods:**

Fifty‐four pregnant women with preeclampsia from October 2017 to June 2018 were included in this study. Twenty‐eight matched healthy pregnant women were set as the control group. The general clinical characteristics were measured. Serum STAT4 and sEng were detected by ELISA. Correlation between STAT4 and sEng, and their diagnostic value in preeclampsia were analyzed.

**Results:**

Compared with control, the prothrombin time in preeclampsia was significantly lower, while the mean arterial pressure, 24‐hour urine protein, serum creatinine, fibrinogen, and ALT were significantly higher. The circulating levels of STAT4 and sEng were significantly increased in the preeclampsia. The serum levels of STAT4 and sEng in preeclampsia were positively correlated. For the diagnosis of preeclampsia by the serum STAT4, AUC is 0.902, and the sensitivity and specificity are 0.893 and 0.929. By the serum sEng, AUC is 0.873, and the sensitivity and specificity are 0.816 and 0.905.

**Conclusion:**

The serum levels of STAT4 and sEng were significantly increased in preeclampsia with disease severity status, which have promise as diagnostic markers in preeclampsia.

## 1 INTRODUCTION

Preeclampsia is a special disease of pregnancy, which occurs after 20 weeks of pregnancy.^1^ It is characterized by hypertension and proteinuria, which can cause serious complications such as cerebral edema, pulmonary edema, cerebral hemorrhage, heart failure, coagulopathy, liver rupture, placental abruption, fetal growth restriction, and fetal death. It is one of the main causes of increased perinatal mortality.^2^ At present, the reported incidence rate in China is 9.4%, and the maternal mortality rate is 4.2/100 000.^3^, ^4^ Preeclampsia and its associated complications directly result in the death of 10%‐15% pregnant women. Patients with preeclampsia are prone to cardiovascular disease, diabetes, and stroke, and the fetus is also at high risk for hypertension, coronary heart disease, dyslipidemia, obesity, impaired glucose tolerance, and type 2 diabetes.^5^

There are many theories about the pathogenesis of preeclampsia, including the theory of immune imbalance, the theory of oxidative stress, the theory of placental ischemia and hypoxia, the theory of the abnormality of trophoblast invasion, the theory of endothelial cell injury, the theory of genetic susceptibility, the theory of renin‐angiotensin‐aldosterone, dysregulation of coagulation and fibrinolysis system, and theory of nutritional deficiencies, insulin resistance, and environmental factors.^6^, ^7^, ^8^, ^9^ These theories are frequently integrated and interrelated with each other. For the understanding of preeclampsia, it is considered to be a placenta‐derived disease, a series of syndromes caused by placental ischemia and hypoxia; it can also be considered as a maternal disease, that is, placental development is normal.^2^, ^6^ However, because the pregnant woman is in a chronic disease state and is susceptible to preeclampsia,^9^ it is now recognized that many of the above factors may lead to placental ischemia and systemic endothelial injury through the same or different ways, which may lead to the occurrence and development of preeclampsia.

Vascular endothelial injury is an important cause of the pathogenesis of vascular disease including pregnancy‐induced hypertension.^10^, ^11^ The imbalance of angiogenesis and anti‐angiogenic factors in the maternal circulation plays a key role in the pathogenesis of preeclampsia. Soluble endoglin (sEng) is an anti‐angiogenic factor, a soluble structure of the extracellular domain structure of Eng, with a molecular weight of 65 k Da.^12^ sEng is also a cell surface co‐receptor of TGF‐β, which is competitive. Binding to circulating TGF‐β1 blocks TGF‐β1 signaling, resulting in increased vascular permeability and inhibition of angiogenesis.^13^ sEng is less secreted in normal tissues and increased in hypoxia. Many studies have shown that sEng is significantly elevated in patients with preeclampsia.^13^, ^14^ Levine et al^15^ showed that the level of sEng in serum began to rise 8‐10 weeks before the onset of preeclampsia symptoms.

Signal transducer and activator of transcription (STAT) regulates the biological behavior of immune cells by mediating extracellular signals of inflammatory mediators and is essential for inflammation.^16^ Studies have shown that, in general, cytokines (ie, sEng), growth factors, etc, can bind to the corresponding receptors on the cell surface, thereby initiating an intracellular tyrosine kinase phosphorylation cascade, which is altered by the action of kinases such as JAK2, MAPK, or mTOR, altering the process of cell metabolism, growth, and immune response.^17^, ^18^, ^19^ STAT protein family is a class of transcription factors discovered in recent years, including seven members, STAT1, STAT2, STAT3, STAT4, STAT5a, STAT5b, and STAT6.^20^ The STAT1 is downregulated in T cells in preeclampsia. The STAT3 activation in placenta was attenuated by sustained hypoxia, which might contribute to the pathogenesis of preeclampsia.^21^ It was demonstrated that STAT4 was significantly higher in placenta of preeclampsia patients than that of normal late pregnant women.^22^

This study analyzed the expression of STAT and sEng in the preeclampsia and normal pregnancy patients, and the relationship between STAT4 and sEng in preeclampsia.

## 2 MATERIALS AND METHODS

### 2.1 Research object

In the period from October 2017 to June 2018, the patient with preeclampsia was admitted to the hospital for diagnosis. During the same period, a certain number of normal pregnant women were randomly selected as the control group. There were 54 patients with preeclampsia, including 28 cases of mild preeclampsia as the mild preeclampsia group and 26 cases of severe preeclampsia as the severe preeclampsia group. Twenty‐eight healthy pregnant women matched with age were used as the control group. The sample size is calculated following the published methodology.^23^ There was no significant difference in general clinical data including age and body mass index between the study group and the control group. This study was conducted in accordance with the Helsinki Declaration and approved by the Ethics Committee of Ningbo Mingzhou Hospital. Informed consent was signed by all the subjects.

### 2.2 Diagnostic criteria

The diagnostic criteria for preeclampsia are as follows: (1) The basic diagnostic criteria for mild preeclampsia are as follows: blood pressure ≥ 140/ 90 mm Hg, urine protein ≥0.3 g/24 hours, or random urine protein +; preeclampsia patients with any of the following adverse conditions can be diagnosed as severe preeclampsia: (a) continuous increase in blood pressure: systolic blood pressure ≥160 mm Hg and/or diastolic blood pressure ≥110 mm Hg; (b) proteinuria ≥2.0 g/24 hours or random proteinuria ≥ (++); (c) serum creatinine ≥1.2 mg/dL unless previously known to have increased; (d) platelets <100 × 10^9^/L; (e) microvascular hemolysis—LDH increase; (f) elevated serum transaminase levels—ALT or AST; (g) persistent headache or other brain or visual impairments; and (h) sustained upper abdominal pain.

The preeclampsia patients were included following the criteria: (a) age 20‐40 years; (b) hypertension, proteinuria, and edema; and (c) singletons, all of which are terminated by cesarean section and are healthy in the past.

The exclusion criteria are as follows: pregnancy with medical complications or other obstetric complications including chronic kidney disease and heart disease that affect the possibility of developing preeclampsia.

### 2.3 Specimen collection and processing

Serum specimens: All subjects were given 3 mL of venous blood on an empty stomach in the morning before the treatment, and the serum was aspirated after 3500 rpm/min for 15 minutes and stored in a refrigerator at −80°C.

### 2.4 Enzyme‐linked immunosorbent assay (ELISA)

The testing method of sEng and STAT4 ELISA kits (R&D SYSTEMS, USA) is described in detail by the manufacturer's kit's instructions. The experimental method is briefly described as follows: All reagents are thoroughly mixed before the test. Do not allow the liquid to generate a large amount of foam, so as to avoid adding a large amount of air bubbles during the loading, resulting in errors in the loading. Take 10 μL of the sample and mix 90 μL of the sample dilution in the sample well. The plate was covered, gently shaken and mixed, incubated at 37°C for 2 hours, and then incubated with antibody (1:100), 100 μL per well, gently shaken and mixed at 37°C for 1 hour. Then, the plate was incubated with streptavidin‐HRP (1:100), 100 μL per well, gently shaken and mixed at 37°C for 1 hour, added 90 μL of substrate to each well, and incubated at 37°C for 30 minutes. The reaction was terminated by 50 μL of termination solution, and the OD value of each well was measured at a wavelength of 450 nm.

### 2.5 Detection and correlation analysis of clinical indicators

The related clinical indexes of the pregnant women in each group were detected, including the prothrombin time (PT), fibrinogen (Fib), 24‐hour urine protein, serum creatinine (SCr), and alanine transaminase (ALT) within a week before delivery. The relationship between serum STAT4 level and pregnant women in the mild and severe preeclampsia groups was also analyzed.

### 2.6 Statistical analysis

Statistical analysis was performed using SPSS l9.0 statistical software. The measurement data were expressed by Mean ± SD. The paired‐sample *t* test was used to compare the prenatal and postnatal groups. The three groups of data were compared with one‐way analysis of variance (ANOVA) with Newman‐Keuls method (the levels of sEng and STAT4 are normally distributed). Pearson’s correlation coefficient was used for correlation analysis. *P* < .05 was considered statistically significant. The receiver operating characteristic curve (ROC curve) was analyzed for STAT4 and sEng, and the area under the curve (AUC), sensitivity, and specificity were obtained.

## 3 RESULTS

### 3.1 General clinical features

Comparison of clinical features of the control group, mild preeclampsia group, and severe preeclampsia group is shown in Table 1. There was no significant difference in age and body mass index among the groups (*P* > .05). The prothrombin time in the mild preeclampsia group and the severe preeclampsia group was significantly lower than that in the normal pregnancy group, while the mean arterial pressure, 24‐hour urine protein, serum creatinine, fibrinogen, and ALT were significantly higher than the control group (*P* < .05).

**Table 1:** Comparison of clinical features of pregnant women in each group

Group	Control	Mild preeclampsia	Severe preeclampsia
N	28	28	26
Age (y)	30.13 ± 2.63	29.39 ± 3.62	31.01 ± 3.25
Body mass index (kg/m^2^)	25.02 ± 1.68	25.98 ± 2.07	25.46 ± 1.94
Mean arterial pressure (mm Hg)	91.25 ± 5.21	114.29 ± 5.47*	125.29 ± 4.93*
24‐h proteinuria (g)	0.07 ± 0.04	0.79 ± 0.83*	5.02 ± 1.63*
Serum creatinine (μmol/L)	71.63 ± 6.72	96.13 ± 9.89*	119.32 ± 10.66*
Prothrombin time (s)	11.02 ± 1.47	9.72 ± 1.24*	8.99 ± 1.46*
Fibrinogen (g/L)	2.68 ± 0.53	3.76 ± 0.82*	4.72 ± 0.83*
ALT (U/L)	27.58 ± 4.72	38.45 ± 8.28*	45.21 ± 10.02*

Note

* *P* < .05 vs control.

### 3.2 The circulating levels of STAT4 and sEng in the serum of patients

The circulating levels of STAT4 were 0.340 ± 0.062, 0.637 ± 0.159, and 1.513 ± 0.182 ng/mL in the control group, mild preeclampsia group, and severe preeclampsia group, with statistically significant difference (*P* < .01). The mild and severe preeclampsia groups were significantly higher than the control group, while the severe preeclampsia group was significantly higher than the mild preeclampsia group (*P* < .01) (Table 2).

**Table 2:** Serum levels of STAT4 and sEng in patients

Group	n	STAT4 (ng/mL)	sEng (ng/mL)
Control	28	0.340 ± 0.062	8.982 ± 1.089
Mild preeclampsia	28	0.637 ± 0.159**	11.421 ± 1.330*
Severe preeclampsia	26	1.513 ± 0.182**△△	13.152 ± 1.735**△
*F*		42.37	29.45
*P*		<.01	<.01

Note

*P < .05, **P < .01 vs control; △*P* < .05, △△*P* < .01 vs mild preeclampsia.

The circulating levels of sEng in serum were 8.982 ± 1.089, 11.421 ± 1.330, and 13.152 ± 1.735 ng/mL in the control group, mild preeclampsia group, and severe preeclampsia group, with statistically significant difference (*P* < .01). The mild and severe preeclampsia groups were significantly higher than the control group, while the severe preeclampsia group was significantly higher than the mild preeclampsia group (*P* < .05) (Table 2). The intra‐ and inter‐assay coefficients of variation were 7.8% and 9.2%, respectively.

### 3.3 The correlation between the expression of STAT4 and sEng in peripheral blood of patients with preeclampsia

**Figure 1:**
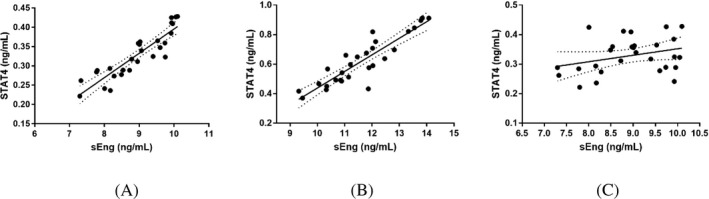
The correlation between the expression of STAT4 and sEng in peripheral blood of patients with preeclampsia. A, STAT4 and sEng in patients with mild preeclampsia. B, STAT4 and sEng in patients with severe preeclampsia. C, STAT4 and sEng in normal subject

The expression of STAT4 and sEng in peripheral blood of patients with mild preeclampsia was positively correlated (*r* = .808, *P* < .001) (Figure 1A). The expression of STAT4 and sEng in peripheral blood of severe preeclampsia was also positively correlated (*r* = .807, *P* < .001) (Figure 1B). However, the expression of STAT4 and sEng in peripheral blood of control subjects was not significantly correlated (Figure 1C).

### 3.4 The diagnosis value of STAT4 and sEng for preeclampsia

**Figure 2:**
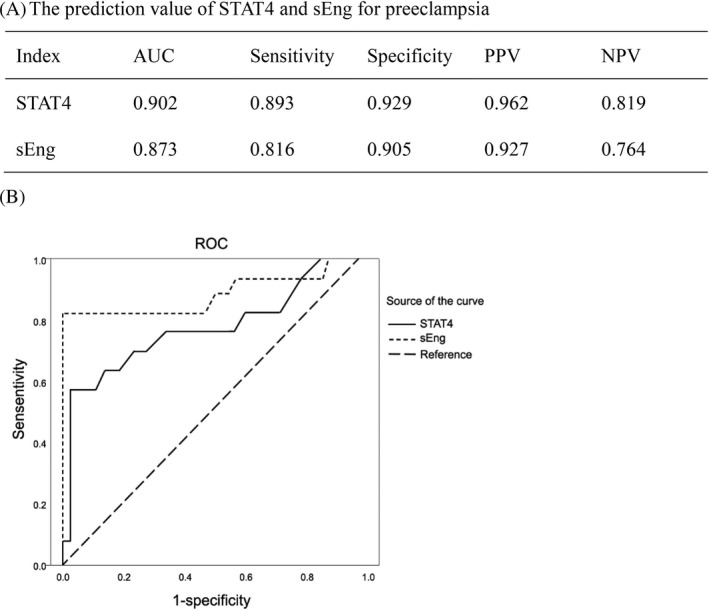
The diagnosis value of STAT4 and sEng for preeclampsia. A, Diagnosis value. AUC, area under curve; NPV, negative predictive value; PPV, positive predictive value. B, ROC curve

For the diagnosis of preeclampsia by the serum STAT4, AUC is 0.902, and the sensitivity and specificity are 0.893 and 0.929, respectively. For the diagnosis of preeclampsia by the serum sEng, AUC is 0.873, and the sensitivity and specificity are 0.816 and 0.905, respectively. The sensitivity and specificity of STAT4 diagnosis were superior to sEng (Figure 2).

## 4 DISCUSSION

Vascular endothelial injury is an important cause of hypertensive disease in pregnancy.^11^ The imbalance of angiogenesis/anti‐angiogenic factors in the maternal circulation plays a key role in the pathogenesis of preeclampsia.^24^, ^25^ The level of sEng in serum increased before the onset of preeclampsia symptoms.^15^ STAT4 was significantly higher in placenta of preeclampsia patients than that of normal late pregnant women.^22^

Here, we reported that STAT4 and sEng involved in preeclampsia.

Preeclampsia is a generalized systemic disease of pregnancy, which occurs after 20 weeks of pregnancy.^1^ The clinical manifestations are characterized by hypertension and proteinuria, accompanied by multiple organ damage. Epidemiological investigation shows that the incidence of preeclampsia is 3%‐5%, which is the main cause of maternal and infant morbidity and mortality.^26^ At present, the etiology theories generally include the theory of uterine‐placental ischemia, theory of abnormal blood vessel‐regulating substance, immunological theory, and hereditary theory.^26^, ^27^ Placental ischemia and hypoxia and endothelial dysfunction are recognized as important factors leading to preeclampsia.^6^, ^7^, ^8^, ^9^ In pregnancy, the fetus is a homologous allograft, so the process of pregnancy is actually autologous allogenic process. Under normal conditions, the maternal immune system is balanced, and the preeclampsia patients have a rejection between the placenta and the mother body, which can lead to the invasion of trophoblastic cells and the uterus.^2^, ^6^ The disorder of disk spiral artery remodeling causes low placental perfusion, ischemic anoxia, and abnormal placental function.^28^ The placenta syntheses and releases a variety of inflammatory mediators and vasoactive factors such as STAT4, sFlt‐l, sEng, TNF‐alpha, and IL‐6, which act on vascular endothelial cells, and the dysfunction of endothelial cells might cause pathological process such as tumorigenesis.^29^ The elevated levels of circulating endothelin (ET‐1) and reactive oxygen species (ROS), as well as increased sensitivity to angiotensin II, lead to multiple organ dysfunction.^12^, ^13^ The typical clinical manifestations of preeclampsia are hypertension, edema, and proteinuria.

sEng is an anti‐angiogenic factor, which has many similar characteristics with sFlt‐1 and is also involved in the pathogenesis of systemic endothelial dysfunction in preeclampsia. The exact relationship between sEng and sFlt‐1 is still not clear.^30^ Studies have shown that 65Da sEng monomer in the placenta of patients with preeclampsia is four times higher than normal pregnant patients.^31^ The mechanism by which sEng participates in the pathophysiological process of preeclampsia is not well understood.^11^, ^32^, ^33^, ^34^ sEng is almost undetectable in non‐pregnant women. In normal pregnant women, it is also a low level in the early stage, and the tendency to increase gradually in the middle and later stages of pregnancy at 36‐38 weeks.^35^ sEng can induce apoptosis of human umbilical vein endothelial cells, and the integrity of the structure and function of blood vessel walls is destroyed.^36^ Sarosh^37^ shows that sEng can be used as an independent marker to measure the level of sEng in serum of suspected preeclampsia patients 34 weeks ago and can predict the occurrence of preeclampsia. In our study, the levels of sEng in the serum of the control group, mild preeclampsia group, and severe preeclampsia group were increased in turn, and the difference was statistically significant. The severe preeclampsia group was higher than the mild preeclampsia group, indicating that high level of sEng is associated with severe preeclampsia.

STAT4 acts as transcription factor and cytokine. STAT4 is a specific transduction factor for the differentiation of Th1 cells. It is a key component of IL‐12/STAT4/INF‐γ signal transduction pathways in various immune regulatory cells, which is essential for the development of Th1 cells with complete functions.^38^ The STAT4 pathway is the main signal transduction pathway for cytokine regulation of immune cell differentiation to Th1.^39^ STAT4‐deficient mice showed an advantage in Th2 immune response.^38^ The high expression of STAT4 can promote the recruitment and overexpression of Th1 and the activation of macrophages, etc, inducing dominant expression of IL‐12 and INF‐γ, which is eventually damage the pregnancy.^40^ Our results suggested that STAT4 involved in preeclampsia and it was closely associated with sEng. In this study, we detected the expression of STAT4 and sEng in the serum of patients with preeclampsia and found increase in STAT4 levels in preeclampsia, which were closely related to the condition of the disease. The more severe the preeclampsia, the higher the STAT4 level. Further analysis of the ROC curve revealed the serum STAT4 AUC is 0.902, and the sensitivity and specificity are 0.893 and 0.929, respectively. For the diagnosis of preeclampsia by the serum sEng, AUC is 0.873, and the sensitivity and specificity are 0.816 and 0.905, respectively. The limitation of this study is the levels of sEng and STAT4 in cases that were diagnosis with preeclampsia versus normal pregnant women. The prediction value of those factors should be studied in the future.

In conclusion, both STAT4 and sEng have diagnosis values for preeclampsia, but the sensitivity and specificity of STAT4 were better than sEng, suggesting STAT4 can be used as a novel serum marker for diagnosis of preeclampsia, and thus postponed the development of the disease.

## CONFLICT OF INTEREST

The authors declare that there is no conflict of interest.

## Data Availability

The data used to support the findings of this study are available from the corresponding author upon request.
